# Supplementary material for: A lncRNA fine tunes the dynamics of a cell state transition involving Lin28, let-7 and de novo DNA methylation
Source: eLife. 2017 Aug 18;6:e23468. doi: 10.7554/eLife.23468 (PMC5562443; doi:10.7554/eLife.23468)
Supplement: Supplementary file 1. — DOI: http://dx.doi.org/10.7554/eLife.23468.019 [file elife-23468-supp1.docx]

**Supplementary File 1: Primers for generating *Epn* Targeting vector and genotyping.**

**1A: Recombineering primers for BAC targeting**

| **Name** | **Sequence** |
| --- | --- |
| miniU | GGTCCCCATGGGCACAAAGGTAATTCCCCAGAGCTTAAAGTATGTTCCTTTAGCATTAGATCCAAAGGAATTCGCCCACTTTCACTTCCGATCATATTCAATAACCCTTA |
| miniD | TCTTCAGGGAGCAGAAGGGTGATGGTCAGTTAATCTTTTAACTACTTCCATGACGTTAAGAGGAGGGGCTAGTCCCTGCCTAAAGATGGGTAGTTCTTTAGACGATGGAT |

**1B: PCR primers for constructing BAC retrival vector**

| **Name1** | **Forward** | **Reverse** |
| --- | --- | --- |
| ReU | AATTACTAGTGTAAAAATGTTTTTCCTGCCTTTCT | AATTAAGCTTGGGAATAACCAATTTTCTGTGTTTA |
| ReD | AATTAAGCTTTATGATAGCTCAACTCTCCCTACCA | AATTCTCGAGCAAGGTCTGTATCATCACCATGTAG |

**1C: Genotyping primers for *Epn* knockout**

| **Name** | **Sequence** |
| --- | --- |
| wtF | AGTCGATCCCTGCTGTTGAAAATC |
| pAF | GAGGATTGGGAAGACAATAGCAG |
| wtR | TTAATGTTTCATTTCAGTCCCTGTT |
| wtUF | CATGAAGGCTAACTTCCATTTCC |
