# Supplementary material for: A lncRNA fine tunes the dynamics of a cell state transition involving Lin28, let-7 and de novo DNA methylation
Source: eLife. 2017 Aug 18;6:e23468. doi: 10.7554/eLife.23468 (PMC5562443; doi:10.7554/eLife.23468)
Supplement: Supplementary file 2. — DOI: http://dx.doi.org/10.7554/eLife.23468.020 [file elife-23468-supp2.docx]

**Supplementary File 2: Primers for generating gRNAs vectors and genotyping for *Dnmt3a/3b* knockouts.**

**2A: DNA oligonucleotides used to generate gRNAs targeting Dnmt3a and Dnmt3b**

| Dnmt3a gRNA1 F | caccgCAGGTTTGGTTGGTCTGTCC |
| --- | --- |
| Dnmt3a gRNA1 R | aaacGGACAGACCAACCAAACCTGc |
| Dnmt3a gRNA2 F | caccgGGACATCTCGCGATTTCTTG |
| Dnmt3a gRNA2 R | aaacCAAGAAATCGCGAGATGTCCc |
| Dnmt3b gRNA1 F | caccgGCAAACTCTCAGGTACTCGA |
| Dnmt3b gRNA1 R | aaacTCGAGTACCTGAGAGTTTGCc |
| Dnmt3b gRNA2 F | caccgACCAGAGCTTGATTTCCGGG |
| Dnmt3b gRNA2 R | aaacCCCGGAAATCAAGCTCTGGTc |

N.B. The capital letters represent the gRNA sequences. The oligoes were annealed and cloned into pX458.

**2B: Genotyping primers for Dnmt3a and Dnmt3b knockout**

| Dnmt3aF1 | AAAGAAGGAAAGAAAGAAGCAAGCA |
| --- | --- |
| Dnmt3aF2 | GACCTCTCCATTGTCAACCCTG |
| Dnmt3aR1 | AGCCATGATTTTCTCTCAGGTATCA |
| Dnmt3bF1 | GTCCTGTCTCTGTTTGATGGAATTG |
| Dnmt3bF2 | AGGGTTCAGAGTCCCTTCTTTC |
| Dnmt3bR1 | CTACATGGTGAAGAGATGAGACCG |
