# Supplementary material for: A lncRNA fine tunes the dynamics of a cell state transition involving Lin28, let-7 and de novo DNA methylation
Source: eLife. 2017 Aug 18;6:e23468. doi: 10.7554/eLife.23468 (PMC5562443; doi:10.7554/eLife.23468)
Supplement: Supplementary file 3. — DOI: http://dx.doi.org/10.7554/eLife.23468.021 [file elife-23468-supp3.docx]

**Supplementary File 3: siRNAs and mature miRNA mimics used in this study.**

| **SiRNA** | **Supplier** | **Catalogue Number** |
| --- | --- | --- |
| Ephemeron A | Qiagen | SI05681977 |
| Ephemeron B | Qiagen | SI05681984 |
| Nanog A | Qiagen | SI01323357 |
| Nanog B | Qiagen | SI04460869 |
| Lin28a A | Qiagen | SI01090327 |
| Lin28a B | Qiagen | SI01090334 |
| Esrrb A | Qiagen | SI02672110 |
| Esrrb B | Qiagen | SI02739569 |
| Tfcp2L1 A | Qiagen | SI01444296 |
| Tfcp2L1 B | Qiagen | SI04401558 |
| Klf2 A | Qiagen | SI01083530 |
| Klf2 B | Qiagen | SI01083544 |
| Klf4 A | Qiagen | SI01083544 |
| Klf4 B | Qiagen | SI001083593 |
| Dnmt3a SMARTpool | Dharmacon | M-065433-01-0005 |
| Dnmt3b SMARTpool | Dharmacon | M-044164-01-0005 |
| Negative control | Qiagen | 1027280 |
| mmu-let-7c-5p mimics | Qiagen | MSY0000523 |
| mmu-let-7g-5p mimics | Qiagen | MSY0000121 |
