# Supplementary material for: A lncRNA fine tunes the dynamics of a cell state transition involving Lin28, let-7 and de novo DNA methylation
Source: eLife. 2017 Aug 18;6:e23468. doi: 10.7554/eLife.23468 (PMC5562443; doi:10.7554/eLife.23468)
Supplement: Supplementary file 4. — DOI: http://dx.doi.org/10.7554/eLife.23468.022 [file elife-23468-supp4.docx]

**Supplementary File 4: Primers for generating Southern and Northern blotting probes by PCR.**

**4A: Primers used for generating Southern blotting probes by PCR for *Epn* targeting validation**

| **Region** | **Forward** | **Reverse** |
| --- | --- | --- |
| 5’ Probe | CTACAAAGAAGAGACACAGGCCTAC | ACAGATTTCTAAAAGACCCCATCAG |
| 3’ Probe | GACCTCCACAGTCCTCTAAGTGTAG | GCTGGCTGTTCTGATTGTACTATTT |

**4B: Primers used for generating northern blotting probes by PCR**

| **Gene Name** | **Forward** | **Reverse** |
| --- | --- | --- |
| Beta-Actin | CAAGAGAGGTATCCTGACCCTGAAG | GTCAGGCAGCTCATAGCTCTTCTC |
| Nanog | AAGGACAGGTTTCAGAAGCAGAAGT | AGAGAAGTTTTGCTGCAACTGTACG |
| Ephemeron | GAGACAGAAAGACCAACACCAGAAA | AGAGAAACCAAGGAGACCAGACAGT |
