# Supplementary material for: A lncRNA fine tunes the dynamics of a cell state transition involving Lin28, let-7 and de novo DNA methylation
Source: eLife. 2017 Aug 18;6:e23468. doi: 10.7554/eLife.23468 (PMC5562443; doi:10.7554/eLife.23468)
Supplement: Supplementary file 5. — DOI: http://dx.doi.org/10.7554/eLife.23468.023 [file elife-23468-supp5.docx]

**Supplementary File 5: Primers for RACE and nested PCR amplification.**

**5A: 5’ RACE and circular nested PCR primers**

| **Name** | **Sequence** |
| --- | --- |
| Reverse Transcription primer | 5'[P]AGAAACCAAGGAGACCAGACA |
| A1 | CCACACCTATTACATGGGGATT |
| A2 | CCAGTCGATCCCTGCTGTTG |
| S1 | TCTTCCCTCATGGTGTCTAGG |
| S2 | TGCTACACACCAGGCTGGAA |

**5B: 5’ RACE and circular nested PCR primers**

| **Name** | **Sequence** |
| --- | --- |
| Reverse Transcription primer | TCTCACACGACTCACGACAGGGCAAGCAGTGGTATCAACGCAGAGTGCTTTTTTTTTTTTTTTTTTTTTTTTTTTVN |
| PCR primer from RT-primer side | AGTGGTATCAACGCAGAGTGC |
| Ephemeron specific primer | ATCTGTAACGGGCTCCCATA |
