# Supplementary material for: A lncRNA fine tunes the dynamics of a cell state transition involving Lin28, let-7 and de novo DNA methylation
Source: eLife. 2017 Aug 18;6:e23468. doi: 10.7554/eLife.23468 (PMC5562443; doi:10.7554/eLife.23468)
Supplement: Supplementary file 6. — DOI: http://dx.doi.org/10.7554/eLife.23468.024 [file elife-23468-supp6.docx]

**Supplementary File 6: Primers for Real-time quantitative RT-PCR.**

| **Gene Name** | **Forward** | **Reverse** |
| --- | --- | --- |
| Actinβ | ACCAGAGGCATACAGGGACA | ACCAGAGGCATACAGGGACA |
| Nanog | AGGGTGTGCTACTGAGATGCTCTG | CAACCACTGGTTTTTCTGCCACCG |
| Lin28a | AGTCTGCCAAGGGTCTGGAA | CGCTCACTCCCAATACAGAACA |
| Dnmt3a | CCGACGATGAGCCTGAGTAT | CTGTCATCCACCAAGACACAA |
| Dnmt3b | CTCGCAAGGTGTGGGCTTTTGTAAC | CTGGGCATCTGTCATCTTTGCACC |
| Epn | TGGTTGGGGAGGAGTAGTTG | GCAGTGCCTAGACACCATGA |
| Klf2 | GGTAGTGGCGGGTAAGCTC | AACTGCGGCAAGACCTACAC |
| Klf4 | CACCATGGACCCGGGCGTGGCTGCCAGAAA | TTAGGCTGTTCTTTTCCGGGGCCACGA |
| Tbx3 | TGTGCCTGTTGGACCATTAGTT | AGCCAGCTCTACTTGAAAGCAT |
| Esrrb | GGCGTTCTTCAAGAGAACCA | CCCACTTTGAGGCATTTCAT |
| Tfcp2l1 | GGGGACTACTCGGAGCATCT | TTCCGATCAGCTCCCTTG |
| Fgf5 | AAAACCTGGTGCACCCTAGA | CATCACATTCCCGAATTAAGC |
| Oct6 | TTTCTCAAGTGTCCCAAGCC | ACCACCTCCTTCTCCAGTTG |
| Otx2 | GACCCGGTACCCAGACATC | GCTCTTCGATTCTTAAACCATACC |
