# Supplementary material for: A lncRNA fine tunes the dynamics of a cell state transition involving Lin28, let-7 and de novo DNA methylation
Source: eLife. 2017 Aug 18;6:e23468. doi: 10.7554/eLife.23468 (PMC5562443; doi:10.7554/eLife.23468)
Supplement: Supplementary file 7. — DOI: http://dx.doi.org/10.7554/eLife.23468.025 [file elife-23468-supp7.docx]

**Supplementary File 7: ChIRP probes used in this study.**

| **Target** | **Sequence** | **Position** |
| --- | --- | --- |
| Scramble | GCTCCCATACATTTCTTCGGCTCTTA[BtnTg] |  |
| 7SK control | ACCTTGAGAGCTTGTTTGGAGG[BtnTg] |  |
| Epn 1 | CTGGTGTGTAGCAGTGCCTAGACAC[BtnTg] | Epn Exon 1 |
| Epn 2 | GTCGATCCCTGCTGTTGAAAATCCA[BtnTg] | Epn Exon 1 |
| Epn 3 | CCAACCACTGAAGGTAATCT[BtnTg] | Epn Exon 1 |
| Epn 4 | CCCTGCTGTTGAAAATCCAA[BtnTg] | Epn Exon 1 |
| Epn 5 | ACCATGAGGGAAGAAATAGA[BtnTg] | Epn Exon 1 |
| Epn 6 | AGATGGCGACCAGAAGGATT[BtnTg] | Epn Exon 2 |
| Epn 7 | CCAGACAGTAGACTCTAACC[BtnTg] | Epn Exon 3 |
| Epn 8 | TTGTATCTCCGTGTGGGTTG[BtnTg] | Epn Exon 3 |
| Epn 9 | TCAGGCATCTTTCAAATGGT[BtnTg] | Epn Exon 3 |
| Epn 10 | CTCACAGTTCGAGGGTTGTT[BtnTg] | Epn Exon 3 |
| Epn 11 | CTCTTGTCTGAAGCTGACAG[BtnTg] | Epn Exon 3 |
| Epn 12 | CTCACAGTTCGAGGGTTGTT[BtnTg] | Epn Exon 3 |
| Epn 13 | CTCTTGTCTGAAGCTGACAG[BtnTg] | Epn Exon 3 |
