# Supplementary material for: A lncRNA fine tunes the dynamics of a cell state transition involving Lin28, let-7 and de novo DNA methylation
Source: eLife. 2017 Aug 18;6:e23468. doi: 10.7554/eLife.23468 (PMC5562443; doi:10.7554/eLife.23468)
Supplement: Supplementary file 8. — DOI: http://dx.doi.org/10.7554/eLife.23468.026 [file elife-23468-supp8.docx]

**Supplementary File 8: Primers used for Lin28a promoter ChIP PCR.**

| **Name** | **Forward** | **Reverse** |
| --- | --- | --- |
| D1 | GGTTCAGAGTACCTCACAAATGC | GAGGAAACAAAGAAGGGCTGTAT |
| D2 | CATTTCTGAAGGTCCAAACCACT | ACATGATAATGATATGGCAAAGG |
| U1 | GTCCGAGTGTATCAACCAAAGTC | AACGTATGAACCTCAAGAGCAAC |
| U2 | CATGGAGCTTTTAACCACAACAG | AAGAGGTCTTGTGTGTGCTCAGT |
| P1 | GGAAACCTGAAGGCTCTACTCTC | TATTCTCCTCAGCTTGGCATTTA |
| P2 | CTGACTTGAGGTCTGCATTGAA | TCATAGGGAGGAGAGAGTGTGAG |
| P3 | GATAGGGAGGGCCAGGTGAT | GCCTCCTCCTGGGTGTCTAT |
| P4 | AGGGTTCCCTAGTCCAGAGATG | TAAATAGAAGGGAGGTGGGAAAG |
