# Supplementary material for: A lncRNA fine tunes the dynamics of a cell state transition involving Lin28, let-7 and de novo DNA methylation
Source: eLife. 2017 Aug 18;6:e23468. doi: 10.7554/eLife.23468 (PMC5562443; doi:10.7554/eLife.23468)
Supplement: Supplementary file 9. — DOI: http://dx.doi.org/10.7554/eLife.23468.027 [file elife-23468-supp9.docx]

**Supplementary File 9: Primers used for Nanog promoter DNA methylation analysis.**

| Region 1outside F | TAGGATAGTTAGGGTTATATAGAGAAA |
| --- | --- |
| Region 1outside R | AAAACCTATATCCTACTTAAAACTC |
| Region 1 inside F | TAGAGTTTTTATATGGGAGATGT |
| Region 1 inside R | AACTACCTTAATAAATTAATCCATACTT |
| Region 2 outside F | AAGGTAATAGAGAAAAATTTGTTTT |
| Region 2 outside R | CTTCCCACAAAAAAAACAAA |
| Region 2 inside F | AAGTATGGATTAATTTATTAAGGTAGTT |
| Region 2 inside R | CCAACCAAATCAACCTATCTAAAA |
